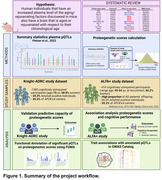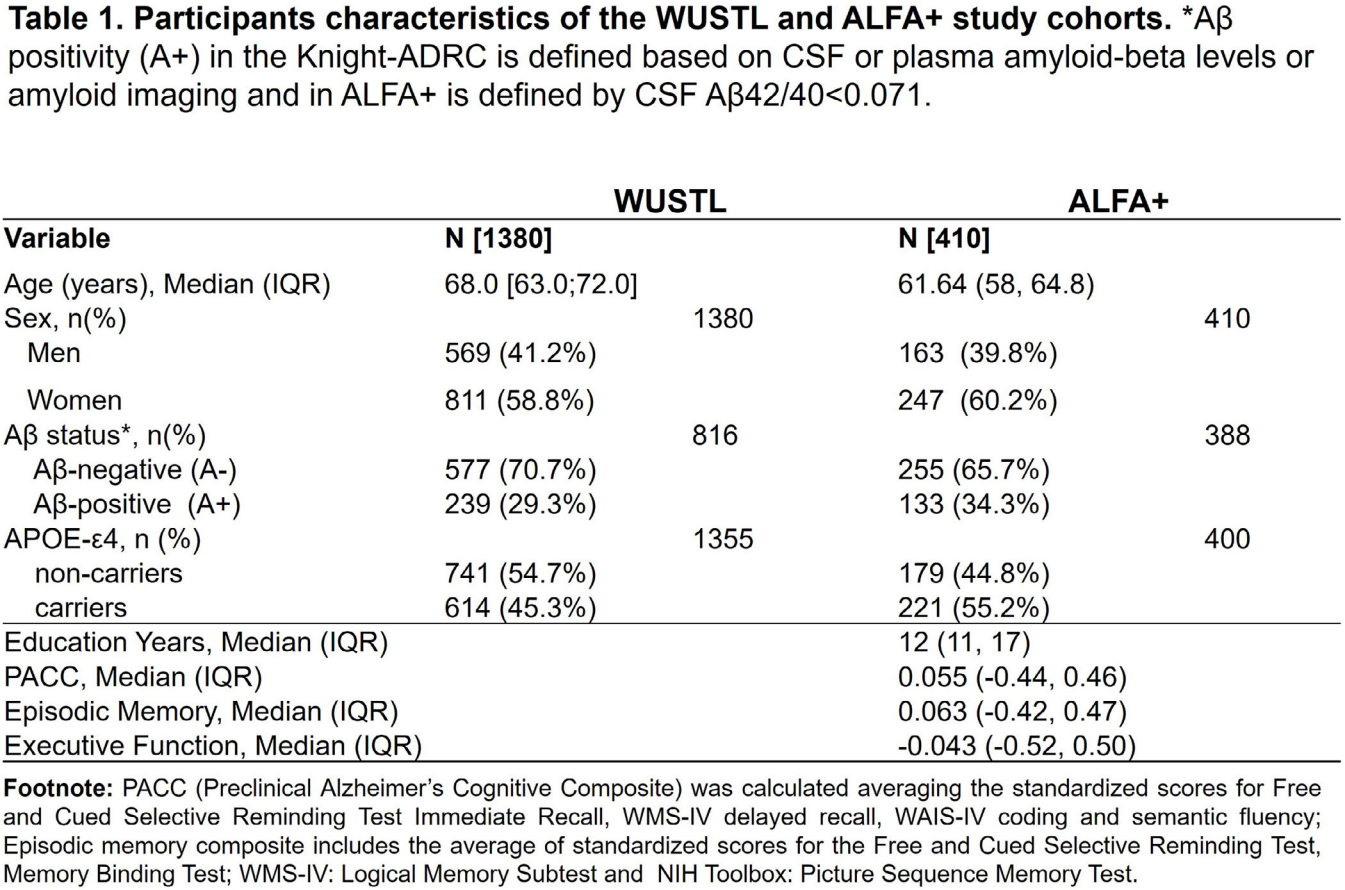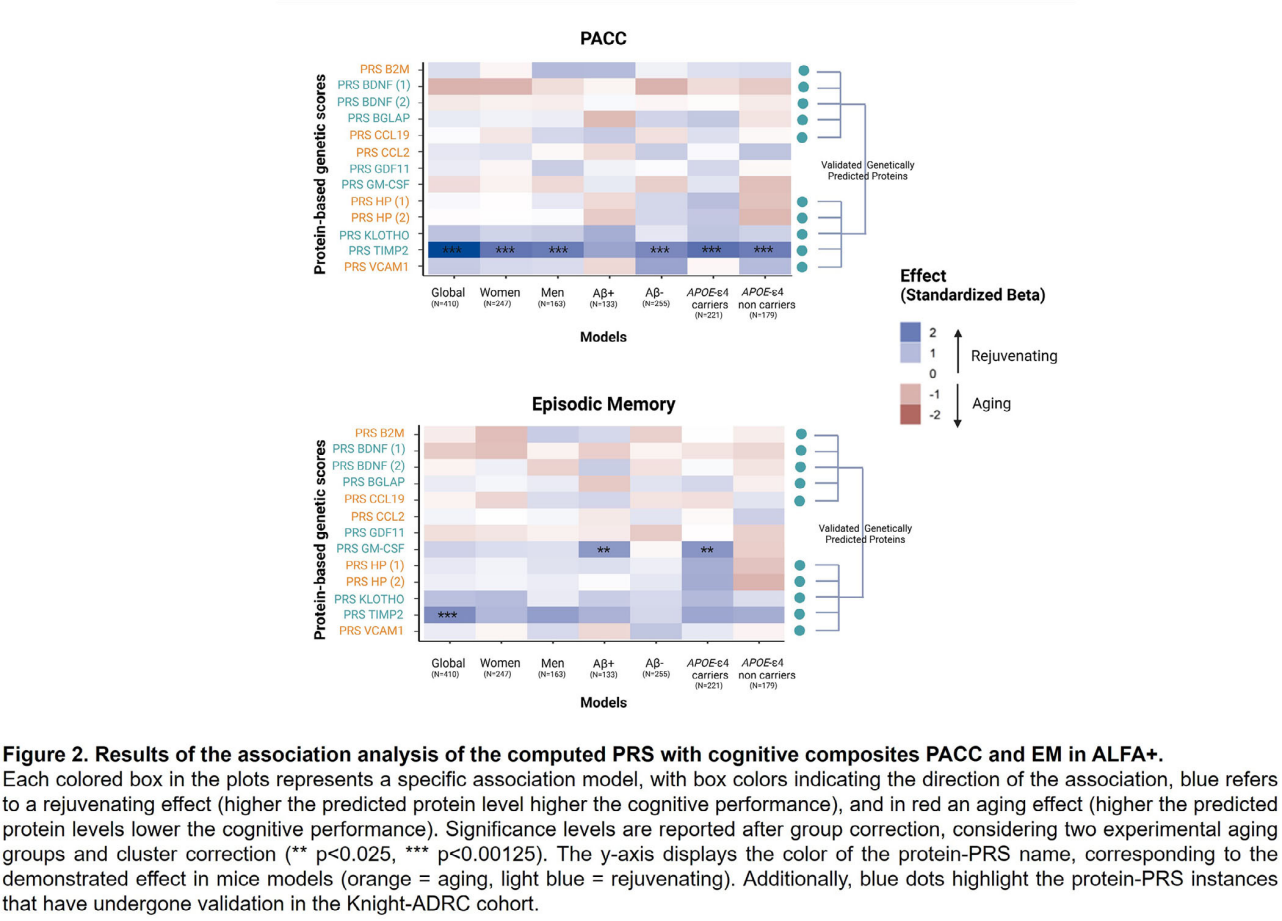# Genetic proxies for predicting plasma protein levels reveal TIMP2 role in human cognitive performance

**DOI:** 10.1002/alz.092194

**Published:** 2025-01-03

**Authors:** Federica Anastasi, Patricia Genius, Blanca Rodríguez‐Fernández, Armand González Escalante, Luis Felipe Hernández‐Villamizar, Luigi Lorenzini, Marta del Campo, Gonzalo Sánchez‐Benavides, Chengran Yang, Jigyasha Timsina, Carolina Minguillón, Manel Esteller, Arcadi Navarro, Carlos Cruchaga, Marc Suarez‐Calvet, Natalia Vilor‐Tejedor

**Affiliations:** ^1^ Barcelonaβeta Brain Research Center (BBRC), Pasqual Maragall Foundation, Barcelona Spain; ^2^ Hospital del Mar Research Institute (IMIM), Barcelona Spain; ^3^ Centre for Genomic Regulation (CRG), Barcelona Institute of Science and Technology (BIST), Barcelona Spain; ^4^ Universitat Pompeu Fabra, Barcelona Spain; ^5^ Departament de Bioquímica i Biologia Molecular, Universitat Autònoma de Barcelona (UAB), Barcelona, Catalonia Spain; ^6^ Department of Radiology and Nuclear Medicine, Amsterdam Neuroscience, Vrije Universiteit Amsterdam, Amsterdam UMC, Amsterdam Netherlands; ^7^ Amsterdam UMC, Amsterdam Netherlands; ^8^ Departamento de Ciencias Farmacéuticas y de la Salud, Facultad de Farmacia, Universidad San Pablo‐CEU, CEU Universities, Urbanización Montepríncipe Spain; ^9^ Centro de Investigación Biomédica en Red de Fragilidad y Envejecimiento Saludable (CIBERFES), Instituto de Salud Carlos III, Madrid Spain; ^10^ NeuroGenomics & Informatics Center, Washington University School of Medicine, St. Louis, MO USA; ^11^ Department of Psychiatry, Washington University School of Medicine, St. Louis, MO USA; ^12^ Barcelonaβeta Brain Research Center (BBRC), Barcelona Spain; ^13^ Institució Catalana de Recerca i Estudis Avançats (ICREA), Barcelona Spain; ^14^ Josep Carreras Leukaemia Research Institute (IJC), Badalona, Barcelona Spain; ^15^ Physiological Sciences Department, School of Medicine and Health Sciences, University of Barcelona (UB), Barcelona, Catalonia Spain; ^16^ Centro de Investigación Biomédica en Red Cancer (CIBERONC), Madrid Spain; ^17^ Institute of Evolutionary Biology (CSIC‐UPF), Department of Experimental and Health Sciences, Universitat Pompeu Fabra, 08003, Barcelona Spain; ^18^ Servei de Neurologia, Hospital del Mar, Barcelona Spain; ^19^ Department of Genetics, Radboud Medical University Center, Nijmegen Netherlands

## Abstract

**Background:**

Murine studies have identified blood proteins that influence brain aging, but translating these findings to humans remains challenging. We used an innovative approach to investigate whether genetically predicted blood levels of proteins linked to brain aging in animal models are associated with cognitive performance in individuals at risk of Alzheimer’s disease (AD) [**Figure 1**].

**Method:**

Through systematic review, we identified 13 circulating proteins with an aging/rejuvenating effect on the mouse brain. We retrieved summary statistics of protein quantitative trait loci (pQTLs) associated with these proteins in human plasma from the Fenland study (Pietzner et al., 2021). We validated their predictive capacity by computing protein‐based genetic scores (protPRS) in 1,380 cognitively unimpaired (CU) individuals from the Knight‐ADRC cohort and analyzing their association with plasma protein levels (measured by Somalogic). We also computed the protPRS for 410 CU individuals at risk for AD from the ALFA+ study (60% women, 55% *APOE‐ε4* carriers; **Table 1**) and assessed their associations with cognitive performance through linear models adjusted by age, sex, and years of education. Stratified models by sex, *APOE‐ε4* carriership, and Aβ status were also assessed. pQTLs included in the significant scores were annotated to explore their biological significance.

**Result:**

Most computed protPRS (10/13) significantly predicted plasma protein levels in the Knight‐ADRC cohort. In ALFA+, we found a significant association between genetic predisposition to elevated plasma TIMP2 (TIMP2‐protPRS) and better cognitive performance (PACC and episodic memory composites). Associations of TIMP2‐protPRS with PACC remained significant in stratified models [**Figure 2**]. TIMP2‐protPRS was associated with the actual plasma TIMP2 levels in ALFA+. The annotated pQTLs included in the TIMP2‐protPRS were associated with traits related with cognition and neuropsychiatric disorders. We also found an age‐dependent expression of genes regulating blood TIMP2 levels in the human brain.

**Conclusion:**

Protein‐based PRS computation may overcome translational challenges encountered in animal studies. Through this method, we showed that genetically predicted levels of plasma TIMP2, known for its rejuvenating effect on mice’s brain, are linked to cognitive performance in CU at risk of AD. This highlights TIMP2 as a potential therapeutic target for age‐related brain diseases.